# Experimental investigation on machining characteristics of titanium processed using electrolyte sonicated µ-ECDM system

**DOI:** 10.1038/s41598-022-20001-4

**Published:** 2022-09-15

**Authors:** K. V. J. Bhargav, P. S. Balaji, Ranjeet Kumar Sahu, Moussa Leblouba

**Affiliations:** 1 Department of Mechanical Engineering, National Institute of Technology Rourkela, Rourkela, 769008 India; 2 Department of Mechanical Engineering, National Institute of Technology Karnataka Surathkal, Mangalore, 575025 India; 3 Department of Civil and Environmental Engineering, University of Sharjah, University City, P.O.BOX 27272, Sharjah, United Arab Emirates

**Keywords:** Aerospace engineering, Mechanical engineering

## Abstract

Micromachining of difficult-to-machine materials is of prime focus nowadays. One such material is Titanium, which has numerous applications in aerospace, chemical, and biomedical industries. The micromachining of Titanium has become the need of the day because of its exhilarating properties. This investigation employs a tailor-made electrolyte sonicated micro-electrochemical discharge machining (ES-µ-ECDM) system to generate microholes in a commercially pure titanium plate with a thickness of 1000 µm. The machining chamber is the ultrasonication unit (36 kHz) with process parameters voltage (V), concentration (wt%), and duty factor (DF) chosen at three levels. The FCC-RSM-based DOE is selected for experimentation to study the machining characteristics like material removal rate, overcut, and circularity. Through holes were achieved at parameters of 80 V, 25 wt%, and 60% DF and 80 V, 30 wt%, and 50% DF. The incorporation of ultrasonication into the system enhanced electrolyte replenishment and evacuation of the debris at the machining vicinity. The assistance technique improved the gas film stabilization around the tool enabling uniform machining. The multi-response optimization is performed using the MOJAYA algorithm to obtain Pareto optimal solutions, and the MADM (R-method) is employed to obtain the optimal parameter. The optimal parameter was found to be 69 V, 30 wt%, and 50% DF, at which the machined microhole was found to have a circularity of 0.9615 with minimal surface defects.

## Introduction

Titanium is an attractive material making it a potential competitor in aerospace, chemical, architecture, and biomedical industries^1^. Due to its properties like high strength-to-weight ratio, corrosion resistance, biological compatibility, cryogenic capabilities, and low density, Titanium is used in numerous applications^2^. Titanium has become quite popular compared to other metallic implant materials because of its remarkable strength and biocompatibility^3^. Despite these advantages, Titanium’s application and alloys are restricted due to their poor machinability. Further, aerospace, gas turbine engines, chemical industry, nuclear, automobile, and medical applications of Titanium require micro holes^4^. The machining of such micro features in Titanium is challenging using the conventional process because of its properties, such as low thermal conductivity and excessive chemical reactivity^5,6^. The low thermal conductivity of Titanium and its alloys causes a surge in cutting temperature near the cutting edge, causing untimely tool failure and surface damage. Because of the strong chemical reactivity, extensive welding of workpiece material occurs at the tooltip, affecting the machining process and increasing manufacturing costs^7^. Furthermore, by making chatter marks on the workpiece during milling, Titanium and its alloys’ machinability were reduced due to its low modulus of elasticity^8^. Machining is a costly operation that accounts for 60% of the overall cost of essential titanium aerospace components^7^.

The limitations mentioned above of conventional micromachining of Titanium can be overcome by modern manufacturing methods like µ-EDM^6^, µ-ECM^9^, and laser beam micromachining^10^. However, these modern methods suffer drawbacks in terms of lower material removal rate with surface damage for µ-EDM^11^ and µ-ECM^12^ and lower circularity for laser with microstructural changes on the workpiece surface^10^. Recently combinations of modern methods have been developed as a hybrid manufacturing process to provide enhanced machining characteristics than their processes. Micro electrochemical discharge machining (µ-ECDM) is a hybrid process that has gained more attraction among people working with difficult-to-machine materials.

The µ-ECDM process is the concoction of µ-ECM and µ-EDM processes, which enhances the material removal rate and accomplishes better surface integrity and dimensional accuracy of the machined surfaces compared to the machined surfaces in most modern or non-traditional subtractive manufacturing processes^13^. Different theories were proposed for the material removal mechanism during the process by the researchers, Ghosh^14^ opposed the theory of the breakdown of the insulating gas mechanism and claimed that the discharging action occurred due to the switching phenomenon. On the other hand, Jalali et al.^15^ have claimed that the ECDM phenomenon is a combination of chemical etching and local heating. Jain et al.^16^ observed some inconsistencies in the theory of Ghosh^14^ and proposed discharge valve theory for the ECDM mechanism. He observed that the discharge characteristics of the ECDM process were similar to arc discharge by Paschen’s curve while Ghosh reported the discharge characteristics to be similar to Townsend discharge by Paschen's curve. Even though there is still a proper theory has not been established to understand the mechanism involved but the understanding from the literature is proposed in this article.

**Figure 1:**
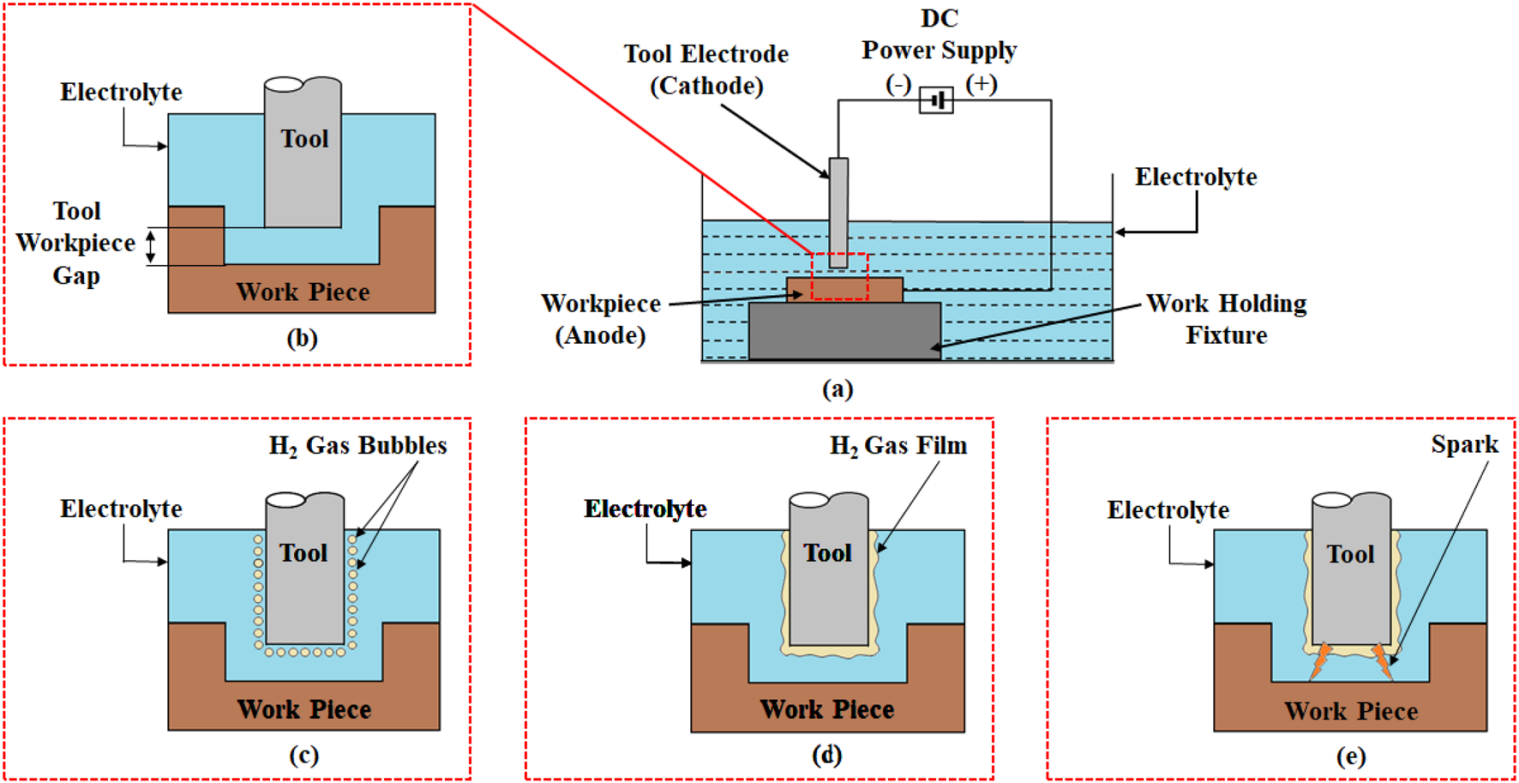
Conventional µ-ECDM process mechanism for conductive workpiece (**a**) schematic (**b**) magnified view of the machining zone (**c**) formation of the gas bubbles (**d**) formation of gas film thickness (**e**) occurance of spark.

The process mechanism of conventional µ-ECDM is shown in Fig. 1. It has a machining chamber with electrolyte in which the workpiece is immersed to a depth of 1–2 mm. If the workpiece is conductive, it is connected to the positive terminal of the DC supply, and the tool is connected to the negative terminal^13,17^. The process initiates with ECM, wherein a series of chemical reactions occur with anodic dissolution. During these chemical processes, hydrogen gas is released at the tool electrode shown in Fig. 1c. This hydrogen gas serves as the dielectric medium that separates the tool electrode and the electrolyte, resulting in a thick gas layer as shown in Fig. 1d. A dielectric breakdown occurs when a critical voltage and a high current density occurz. As a result, the electrons at a high velocity move toward the workpiece, causing a spark as shown in Fig. 1e. This spark is responsible for material removal due to melting and vaporization^18–20^. The combined action of anodic dissolution, melting, and vapourization helps higher material removal.

Electric discharge in the electrolyte is induced by the electrical breakdown of the vapor–gas layer, which results in a significant number of craters on the workpiece surface. Concurrently, electrochemical dissolution reduces these surface imperfections (roughness caused by craters). As a result, the material removal rate is much higher than ECM/EDM, and the surface polish is of superior quality. This technology has the ability to machine electrically conductive materials at a pace five to fifty times faster than ECM and EDM and with improved dimensional precision and surface polish^21,22^.

The disadvantage of the basic µ-ECDM process is machining beyond a certain depth. The researchers have classified material drilling into two regions, one being the discharge regime up to a machining depth of 300 µm with enough electrolyte availability for spark formation, and the other being the hydrodynamic regime, i.e., beyond 300 µm, where replenishment of electrolyte is a grave concern^23,24^. The electrolyte will not reach the bottom of the tool electrode, this will lead to side sparking increasing the hole overcut and protrusions at the bottom surface of the machined channel or hole producing an improper surface finish^25^.

These issues can be resolved using several µ-ECDM process aids, including tool rotation^26–29^, tool sonication^30,31^, workpiece sonication^32^, nanoparticles and surfactants^33,34^, magnetic fields^35^, laser^23^, among others. The following section focuses on a few researches that have been carried out on different materials using ECM and EDM followed by research carried out using ECDM.

Applying the scale effect, Mouliprashanth and Hariharan^36^ carried out electrochemical micromachining. The input parameters voltage, duty ratio, and tool feed rate were varied independently in these studies. Using variations in the pace of material removal, the degree of circularity, and the amount of overcut, they investigated the scale impact at several scales of severity. Similarity theory allowed them to see the scale impact by comparing the granularity of micro and macro systems. In a quantitative analysis of the scale effects, the degree of similarity accuracy was shown to be higher. Anasane and Bhattacharya^37^ machined titanium using the ECM process with seven different electrolytes to investigate the anodic dissolution. Among the seven electrolytes chosen five have shown better anodic dissolution. Out of the five three were aqueous electrolytes of the acid, base, and salt whereas the remaining two were non-aqueous base solutions. It was found that the non-aqueous base solution (ethylene glycol with sodium bromide) produced better results and can be considered a cost-effective and eco-friendly electrolyte for machining titanium using ECMM.

Mouliprashanth and Hariharan's^38^ research helps to determine and evaluate the geometrical and performance characteristics of ECMM on SS304 alloy under the influence of three distinct kinds of electrolytes: non-passivating, passivating, and composite (CPE). Circularity, conicity MRR, and overcut are geometrical and performance characteristics. The results showed composite electrolyte results were better. The Operandi of optimization with the technique with TOPSIS approach for multiresponse characteristics based on MCDM for ECMM process was also performed to obtain optimal process parameters.

Azhar et al.^39^ performed EDM micromachining on three different materials namely aluminum, copper, and stainless steel. They proposed a model (CANFIS) to predict multiple µEDM performances. This model took properties of the material (thermal conductivity, melting point, and electrical resistivity) into consideration to predict MRR, total discharge pulse, overcut, and taperness with more than eighty percent accuracy. Gong et al.^40^ performed EDM milling of sintered NdFeB and studied its effect on surface morphology, cross-section, MRR, surface roughness, and electrode wear rate. Pulse current, pulse interval, and pulse duration were taken as process parameters. The EDM milling was compared with EDM and it was observed that EDM milling had better performance on machined surfaces. EDM milling had a large crater radial size, lower surface roughness, thinner recast layer, and less heat-affected zone. The impact of boron carbide B4C powder blended with dielectric fluid on MRR, EWR, and Ra was experimentally investigated by Kolli and Kumar^41^. The addition of B4C powder to the dielectric fluid resulted in a significant increase in the MRR, but a reduced surface finish. Additionally, at high B4C powder concentrations, adhering phenomena were noticed on the machined surface.

Most of the research using the µ-ECDM process focuses on non-conducting materials, and only a few pieces of literature are available for micromachining conductive materials^11^. The following section focuses on the works of literature available on the micromachining of different materials using the µ-ECDM process, particularly conducting materials.

The machining of non-conducting materials has been explored to a vast extent; hence, only a few pieces of literature are discussed in this section, followed by literature corresponding to conducting materials. Torabi and Razfar^42^ investigated to examine the ECDM process’s potential for the generation of microchannels on polydimethylsiloxane (PDMS). The ECDM technique was capable of fabricating channels on PDMS with greater depth but poor surface quality. Nevertheless, with a reduction in tool diameter, there was a reduction in breadth and surface roughness. Furthermore, grooved tools have produced better outcomes than basic tools. Rathore and Dvivedi^30^ used an ECDM process with a sonicated tool on borosilicate glass to further examine the processing capacity. With the tool’s sonication, the machining quality was increased, and the thickness of the gas film was better managed. While the MRR rose by 11.13% and penetration depth by 27.17% compared to the standard ECDM process, the reduction in hole overcut was 23.10%.

Oza et al.^43^ performed machining on quartz using traveling wire electrochemical discharge machining using zinc coated brass wire. Electrolyte concentration, wire speed, and applied voltage were taken as input parameters. Taguchi's based L9 orthogonal array was used to design the experiments Signal-to-noise ratio and ANOVA were used for the optimization of MRR and kerf width. The findings showed that coated brass wire enhances surface finish and reduces wire breakage. Nema et al.^44^ performed machining on quartz material using the ECDM process. A tungsten tool was used to generate micro holes. Feed rate, electrolyte concentration, and voltage were considered as control parameters while objective parameters were maximizing material removal rate and minimizing tool wear. A heat transfer search algorithm was used for optimizing the process parameters to obtain a set of Pareto optimal solutions.

Kumar et al.^45^ investigated wire ECDM process parameters for machining of quartz material using the Taguchi-based optimization method. According to the findings of the experiments, the zinc-coated brass wire with a 150 µm diameter produced the highest material removal rate and produced the best surface quality. Machining quartz beneath a zinc-coated brass wire may improve surface quality features and the rate at which material is removed. Oza et al.^46^ worked on magnetohydrodynamics to improve electrolyte flushing. Microslits were fabricated using magnetic assisted traveling wire ECDM. The magnets generated Lorentz force which applied torque on electrolyte and resulted in a stirring effect. This increased MRR and cutting length by 85.28% and 48.86%, respectively, and reduced the surface roughness by 30.39%.

Huang et al.^47^ used the ECDM approach to evaluate the effects of rotating tools (high-speed) on tool electrodes and stainless steel workpieces. Burr-free holes were machined when the TWR (axial) decreased as the tool rotation speed increased. Zhang et al.^48^ carried out a comparative study by machining nickel-based superalloy using EDM and Electrochemical Discharge Drilling (ECDD). They investigated the machined holes’ surface integrity (surface topography, surface profile, recast layer, surface roughness, etc.). The authors concluded that the surface integrity improved with ECDD with no recast layer and melted debris. Kumar and Das^11^ compared the machining of titanium alloy using the EDM and ECSM processes. The MRR and surface integrity of the machined holes were evaluated and found that the MRR and the surface topography were better for ECSM than the EDM process. Also, a thicker recast layer and HAZ were observed using EDM. The crater marks, pit holes, and globules were observed to be minimum in the case of ECSM as compared to EDM. Singh et al.^26^ employed the µ-ECDM method with a rotating tool mode, metal matrix composites were machined. It was discovered that the L/D ratio was 23.07 times greater than the µ-ECDM without tool rotation. The study found that 900 rpm was the optimum tool rotation for maintaining a consistent gas film thickness.

The above literature on ECM and EDM shows the possibility of machining conducting materials with their assistance techniques to improve the machining characteristics. Though µ-ECM and µ-EDM have proven their existence in machining conducting materials the process possesses some merits and demerits which are discussed earlier in this section. The demerits can be addressed by processing the materials using µ-ECDM^12^. The review of the literature using the µ-ECDM process suggests that very few studies have been explored in the area of micromachining of conducting materials, and most of the studies are carried out to understand the surface integrity of the machined surfaces, and the literature lacks an understanding of the machining characteristics such as MRR, OC, circularity, etc. Though the µ-ECDM process has the potential to improve the machining characteristics, it lacks machining beyond 300 µm because of inadequate electrolyte supply at the machining zone. Researchers have suggested some assistance techniques to overcome this complexity. One such assistance is electrolyte ultrasonication, which can replenish electrolyte in the machining zone and enhances the brighter prospects of the µ-ECDM process for machining exotic materials. The main objective of the proposed study is the micromachining of a commercially pure titanium plate of 1000 µm thickness using a tailor-made Electrolyte sonicated µ-ECDM (ES-µ-ECDM) system and optimizing the process parameters for maximizing MRR and circularity and minimizing OC using the MOJAYA algorithm coupled with MADM (R-method).

## ES-µ-ECDM prototype

**Figure 2:**
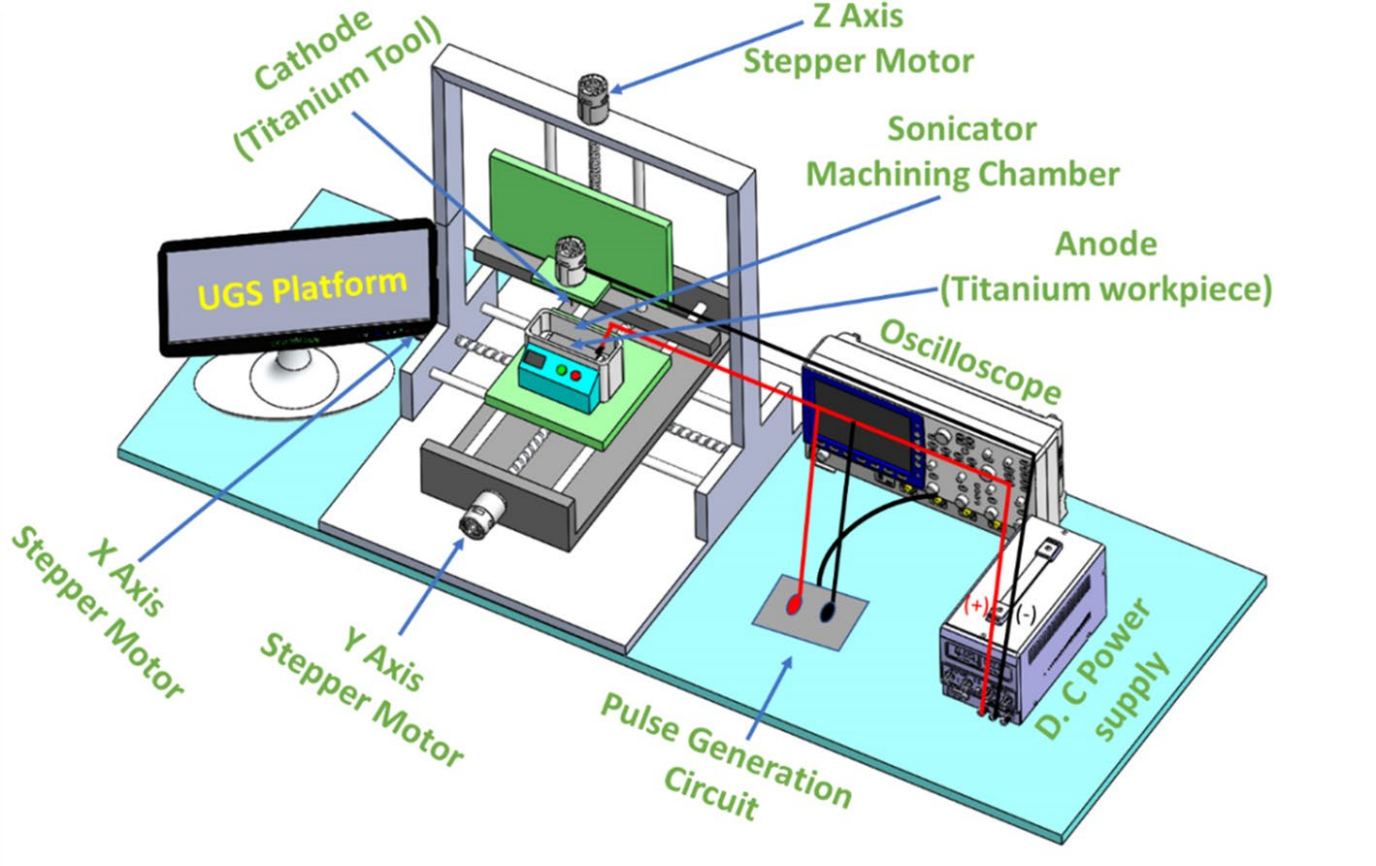
3D schematic view of the developed ES-µ-ECDM experimental system.

**Figure 3:**
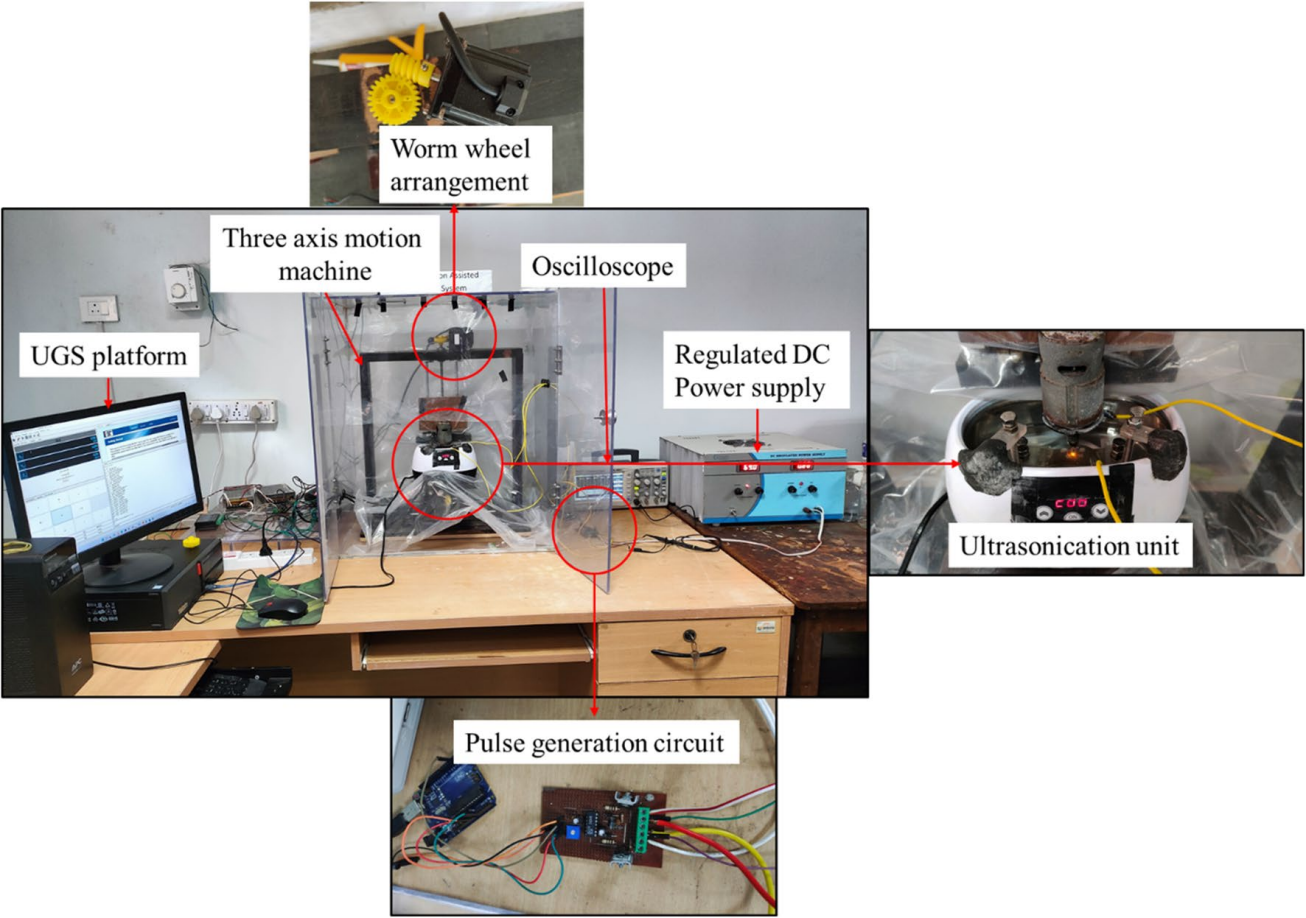
Photographic view of the in-house developed ES-µ-ECDM experimental system.

The in-house developed ES-µ-ECDM system is used to carry out the experiments. The 3D schematic view and photographic view of the ES-µ-ECDM system are shown in Fig. 2 and Fig. 3. The system consists of a 36 kHz frequency ultra-sonication chamber used as a machining chamber with fixtures assembled to hold the workpiece. A regulated DC power supply (0–120 V and 0–5 A) is employed in the system with a pulse generation circuit assembled along with it. The pulse generation circuit helps in producing pulsating DC of the required frequency and duty factor (0–100%). A digital storage oscilloscope is connected to the pulse generation circuit to understand whether the required frequency and duty factor are attained or not. The developed system also consists of a three-axis motion machine driven by stepper motors connected to stepper motor drivers, controlled by a universal g-code sender (UGS) coded using Arduino UNO. The SMPS power sources provide 12 V constant DC to stepper motors, Arduino UNO, and exhaust fan. A worm wheel and worm arrangement are also aligned to the axis of the stepper motor to reduce the feed rate.

## Experimental procedure

A detailed experimental study is conducted to understand the effects of the process parameters on the machining characteristics. In-depth pilot experiments are performed to finalize machining process conditions to accomplish the present task. A commercially pure titanium workpiece of 1000 µm thickness is machined with a titanium tool of 800 µm diameter in NaOH solution. The NaOH electrolyte has a conductivity of 414 mS/cm at a concentration of 20 wt% which reduces to 292 mS/cm with the increase in concentration to 30 wt% at 25 °C. Though at room temperature the conductivity is less during the ECDM process the conductivity increases with rising in temperature as it is known that the kinetic energy of the particles increases^49^. The tool and workpiece are cleaned using acetone to remove the impurities and dirt on the surfaces. The tool is connected to the negative terminal, and the conductive workpiece is connected to the positive terminal. During the machining, material removal usually occurs due to the combined principle of anodic dissolution and melting and vaporization of the workpiece material.

### Pilot experiments

The experiment was conducted initially with 20 wt% concentration and 50% DF, and the voltage was kept increasing till the spark occurred, and it was observed that the spark took place at a voltage of 35–37 V. The voltage was set to 40 V, and machining was conducted. It was observed that there was no material removal, and the tool bend when it came in contact with the workpiece showed only a heat spot on the surface of the workpiece. The voltage was then increased until an appreciable material removal was achieved, which was found to occur at 60 V. Keeping the concentration and voltage constant at 20 wt% and 60 V and reducing the DF to 40% DF led to material removal occurring along with tool bending, which is not desirable as it affects the circularity and taperness of the machined microhole. As a result, the lower level of the process parameters is fixed to 60 V, 20 wt%, and 50% DF.

Then the concentration was increased to 30 wt%, and the DF was kept constant at 50% DF. The voltage was also increased to 70 V and 80 V, which enabled higher material removal and higher overcut. The increase in voltage above 80 V resulted in lower MRR because of the melting of the tool (axial wear). The higher voltage level was fixed to 80 V; now, the concentration was increased to 35 wt%, which resulted in higher electrolyte fumes with higher TWR, which is not desirable. The higher level of concentration and voltage was set to 30 wt% with 80 V. Now, keeping concentration and voltage constant at 30 wt% and 80 V, the DF was increased to 60% DF and then 70% DF. At 70% DF, there was enough material removal with acceptable TWR, and above 70% DF, higher axial tool wear reduced the MRR. Hence the higher level of the factor was set at 80 V, 30 wt%, and 70% DF.

From the pilot experiment and the extensive literature review, the variable and constant process parameters for a detailed experimental study were finalized and tabulated in Table 1.

**Table 1:** Parametric settings for the experiment.

Input parameters	Levels
**Variable parameters**	
Voltage (V)	60, 70, 80
Concentration (wt%)	20, 25, 30
Duty factor (DF) in %	50, 60, 70
**Constant parameters**	
Electrolyte	NaOH
Pulse frequency (kHz)	8
Ultra-sonication frequency (kHz)	36
Workpiece material	Titanium plate of 1000 µm thickness
Tool material	Titanium
Tool diameter (µm)	800
Feed rate (µm/s)	4

**Figure 4:**
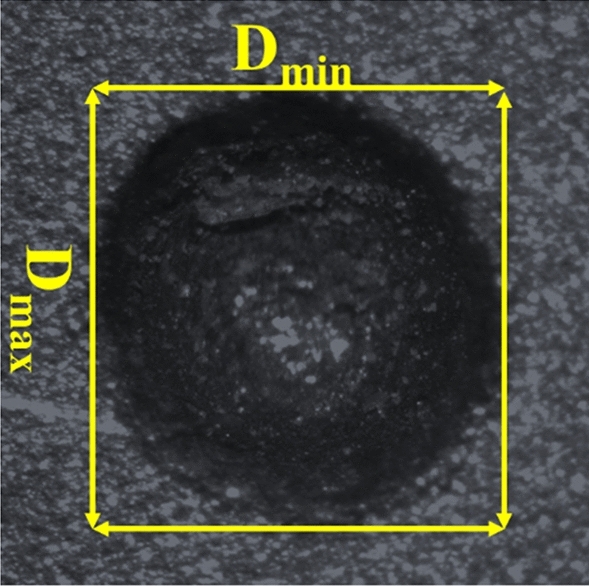
Estimation of circularity using ${D}_{\mathrm{min}}$ and ${D}_{\mathrm{max}}$.

A face-centered central composite RSM (FCC-RSM) design of experiments (DOE) was chosen to carry out the experiments. A three-level three-factor experimental setting has 3^3^ = 27 experiments. The full factorial DOE of FCC-RSM enables the experimental layout with 20 experimental runs (14 axial points and six central points). The machining characteristics are MRR, overcut (OC), and circularity. These are used in the performance evaluation of the process. A multi-objective metaheuristic optimization was carried out using the MOJAYA algorithm paired with the MADM (R-method) to acquire the best process parameters. This optimization aimed to maximize MRR and circularity while reducing OC. The MRR was estimated using a weighing balance (Make: Mettler Toledo) by measuring the weights before and after the machining using Eq. (1). The OC and circularity were estimated using an optical microscope with image processing software (ZEISS Stemi 2000-C). The circularity at the entrance of the machined holes is measured as the ratio of minimum to maximum Feret diameter. Figure 4 illustrates the methodology involved in calculating the circularity at the entrance. The equation Eq. (2) and Eq. (3) is used to measure the OC and circularity. The surface morphologies of the machined microholes were analyzed using a scanning electron microscope (JEOL JSM-6480 LV, EDS: Oxford Instruments).

1 $$ MRR({\upmu }g/\min ) = \left( {\frac{{M_{b} - M_{a} }}{T}} \right) $$

Mb = weight before machining, Ma = weight after machining, and T = machining time

2 $$ OC(\mu m) = (E_{d} - T_{d} ) $$

Ed is the machined hole's entry diameter; Td is the tool's diameter.

3 $$ Cir = \frac{{D_{\min } }}{{D_{\max } }} $$

where D_min_ and D_max_ are the minimum and maximum Feret diameters of machined holes at the entrance.

## Results and discussion

The machining characteristics corresponding to the FCC-RSM layout are tabulated in Table 2. The machining was conducted two times, and the average values were considered.

**Table 2:** FCC-RSM parametric layout with machining characteristics.

S.no.	Voltage (V)	Concentration (wt%)	Duty factor (%)	MRR (µg/min)	OC (µm)	Circularity
1	80	25	60	802.5	542.81	0.90856
2	70	25	60	542	445.12	0.92159
3	70	20	60	510	466.33	0.93205
4	60	25	60	380	310.07	0.86546
5	80	30	50	832.5	585.51	0.96789
6	60	20	70	382.5	338.41	0.86542
7	70	25	60	586.5	459.76	0.91924
8	70	25	60	532.5	442.81	0.92165
9	60	30	70	448	355.75	0.89726
10	60	20	50	357.5	293.47	0.9272
11	70	25	70	555	454.42	0.90307
12	80	20	70	695	534.51	0.91664
13	80	20	50	649	510.4	0.93129
14	80	30	70	777.5	577.21	0.96406
15	70	25	60	557.5	433.97	0.91352
16	70	25	50	547.5	399.746	0.94217
17	60	30	50	412.5	378.43	0.94745
18	70	25	60	568.5	466.65	0.92125
19	70	30	60	582.5	480.97	0.9667
20	70	25	60	552	440.55	0.91386

### Material removal mechanism in ES-µ-ECDM system

**Figure 5:**
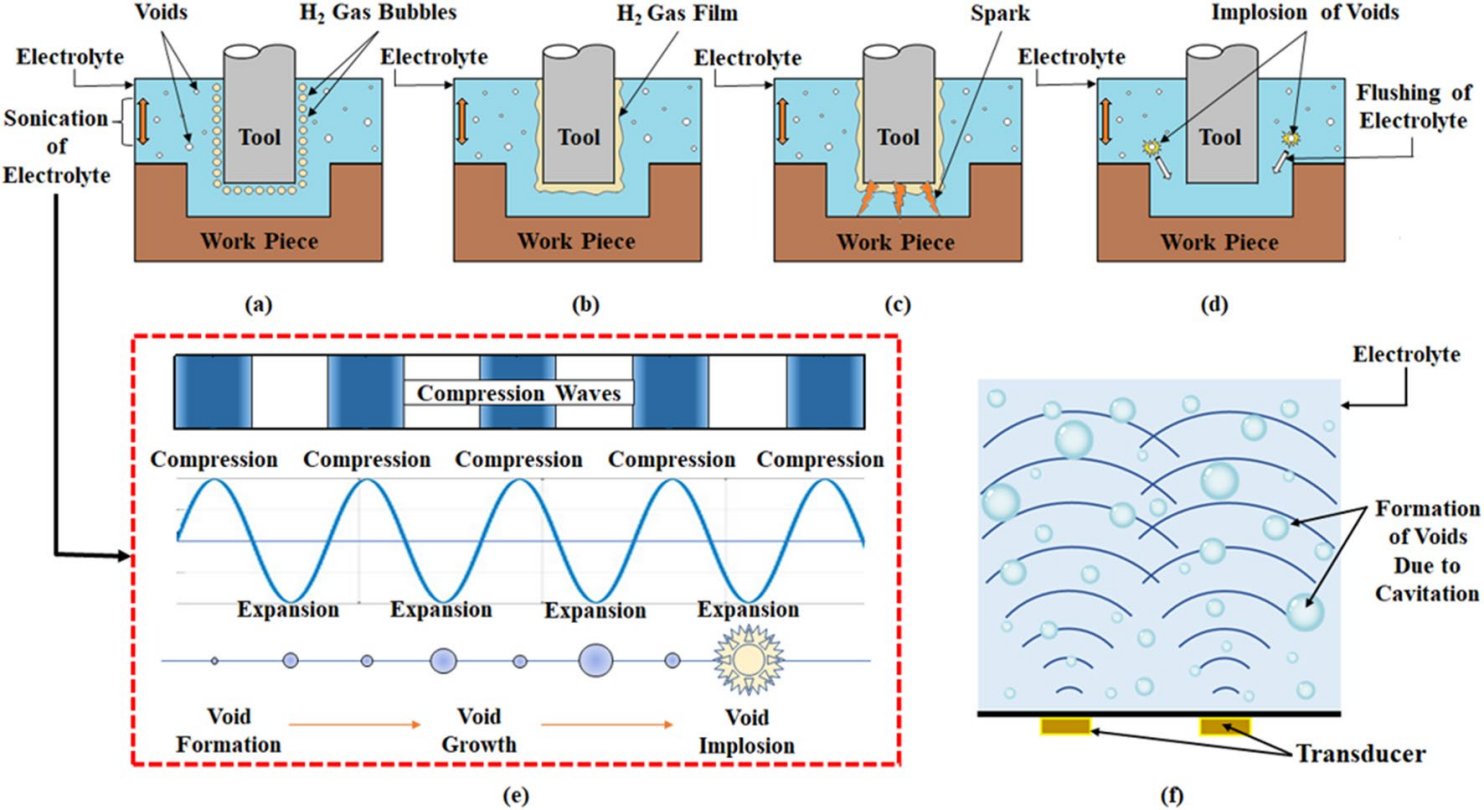
Material removal mechanism of ES-µ-ECDM system (**a**) formation of gas bubbles (**b**) formation of gas film thickness (**c**) occurance of spark (**d**) electrolyte flushining due implosion of the voids (**e**) gradual growth and void implosion phenomenon (**f**) formation of voids due to cavitation.

The detailed material removal mechanism involved in the ES-µ-ECDM system is shown in Fig. 5. The ultrasonication unit is used as an electrolyte chamber where the base plate of the ultrasonicator is attached to a transducer. The transducer provides very subtle vibrations when connected to the power supply.

The motion of the base plate has two strokes. The downward stroke (rarefaction) of the base plate creates low-pressure zones inside the electrolyte fluid. These low-pressure zones initiate the boiling of electrolyte resulting in micro-size gas bubbles (voids) because of the cavitation phenomenon as shown in Fig. 5f ^50^. The voids created due to the downward stroke of the base plate are of microscopic scale and cannot be seen during operation. During the upward stroke (compression), the pressure in those regions increases and reduces the size of the gas bubbles. After passing through several cycles of compression and rarefaction the bubbles tend to grow in size reaching an unstable state as shown in Fig. 5e. At this state on the next compression stroke, the bubble implodes at a high localized jet velocity^51^. This phenomenon is continued in the electrolyte during the whole process but its effect is visualized during pulse off time of the machining. The discharge action takes place during the pulse on time and soon after discharge there becomes a zone of vacuum with very low pressure. During this time, the constant implosion of the voids near the machining zone helps in flushing the electrolyte with high velocity because of the higher pressure difference created. This enables the removal of debris from the machining vicinity and also produces fresh electrolyte for the process to continue. Figure 5a–d explains the procedure of generation of the spark which is quite similar to the conventional µ-ECDM process. In the conventional µ-ECDM process after the hydrodynamic region is reached due to the lack of the electrolyte only side sparking occurs which increases the entrance hole overcut but does not aid in the depth of penetration and also protrusions are formed at the bottom but Fig. 5c clearly shows that sparking occurs at the bottom of the tool surface along with side sparking enhancing the depth of penetration.

### Influence of voltage on machining characteristics

**Figure 6:**
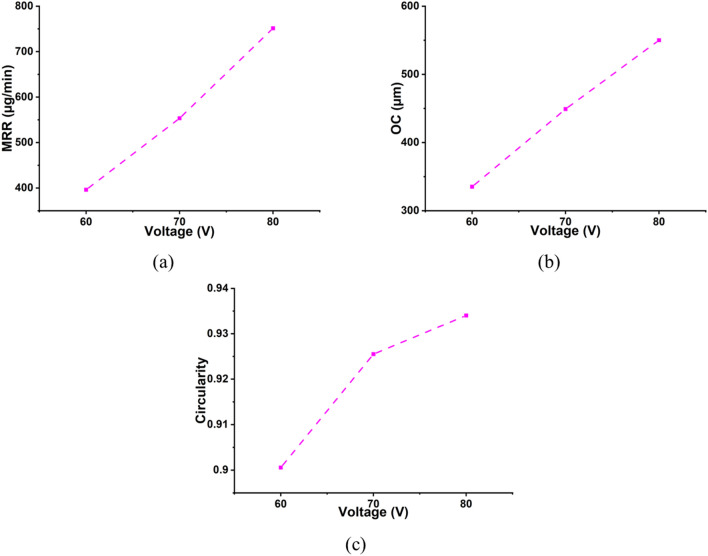
Influence of voltage on machining characteristics (**a**) MRR, (**b**) OC, and (**c**) Circularity.

**Figure 7:**
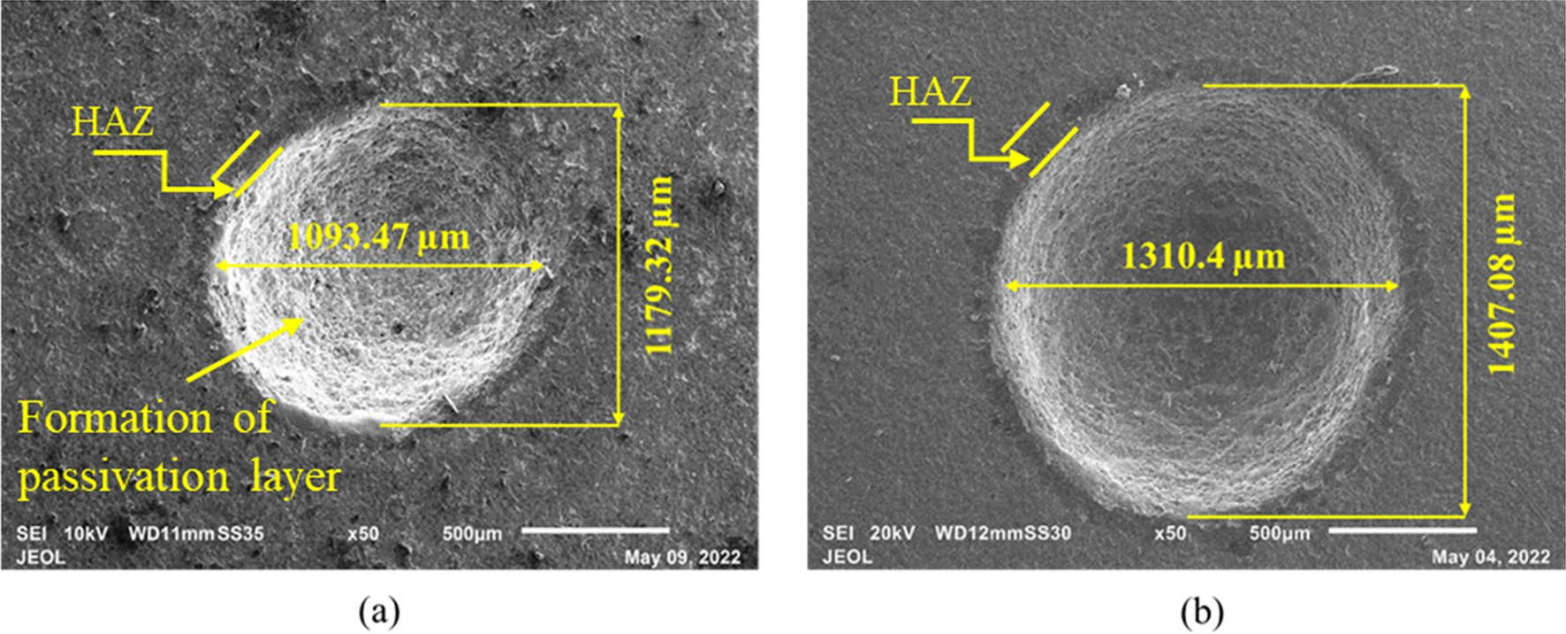
Micrographs of the machined microholes at (**a**) 60 V, 20 wt%, 50% DF and (**b**) 80 V, 20 wt%, 50% DF.

The ES-µ-ECDM system is used for the sonication of the electrolyte, and voltage plays a prominent role in the micromachining of commercially pure titanium workpieces. Stabilizing the spark and spark intensity depends on the gas film’s thickness and voltage, aiding assistances like sonication^52^. The effect of voltage on the machining characteristics is shown in Fig. 6. It can be observed that increasing the voltage from 60 to 80 V increased all the machining characteristics. Ultrasonication provided to the electrolyte helped reduce the gas bubble’s departure radius, resulting in gas bubbles with a thin and more stable gas layer. The latter leads to the channelized spark under the tooltip and thus, increasing the depth of penetration and generating through-holes at higher levels of voltage. The rise in voltage increased the spark intensity at the machining zone enabling a higher MRR. As a result of the increase in voltage from 60 to 80 V, an average percentage increase in MRR of 89.67%. The increase in spark intensity also increases the OC and circularity associated with the machined hole. The percentage increase in OC and circularity with an increase in voltage from 60 to 80 V, keeping concentration and duty factor constant at 20 wt% and 50% DF, is 73.91% and 0.44%, respectively. The average percentage increase in OC and circularity with a surge in voltage is 64.09% and 3.71%, respectively. The SEM images in Fig. 7 show the rise in OC and circularity. As the spark intensity increases, there is an evident increase in OC, but the increase in hole circularity might be due to proper melting and vaporization of the workpiece at higher voltages. The rise in OC also states the evidence for a rise in MRR.

It is also observed that with an increase in voltage the HAZ increases but the formation of the passivation layer was seen to be less. This might be because higher spark intensity due to a surge in voltage increased the HAZ and does not allow the formation of a passivation layer. As the spark hindered the formation of the passivation layer the depth of penetration increased and through-holes are achieved at a higher voltage of 80 V.

### Influence of electrolyte concentration on machining characteristics

**Figure 8:**
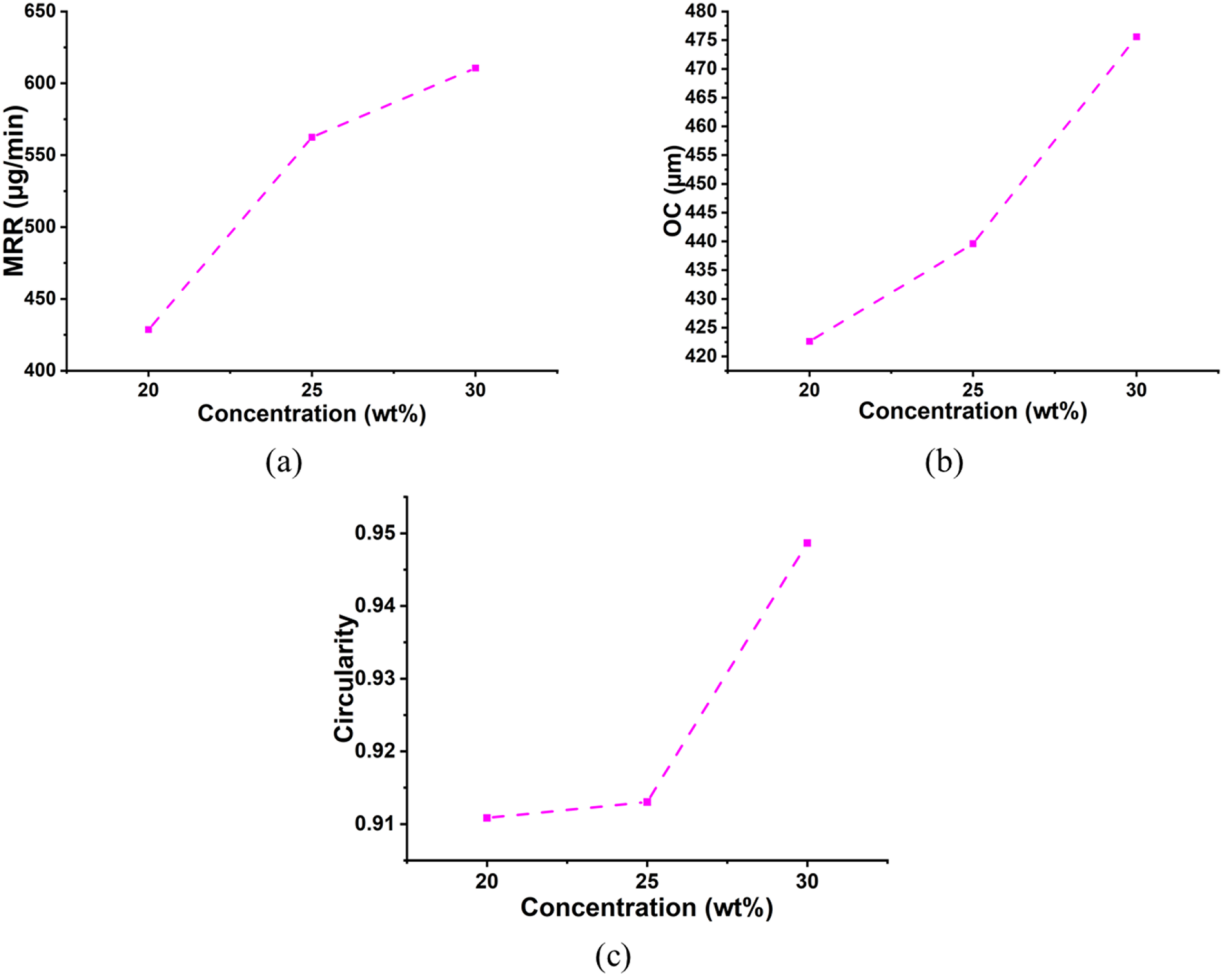
Influence of electrolyte concentration on machining characteristics (**a**) MRR, (**b**) OC, and (**c**) Circularity.

**Figure 9:**
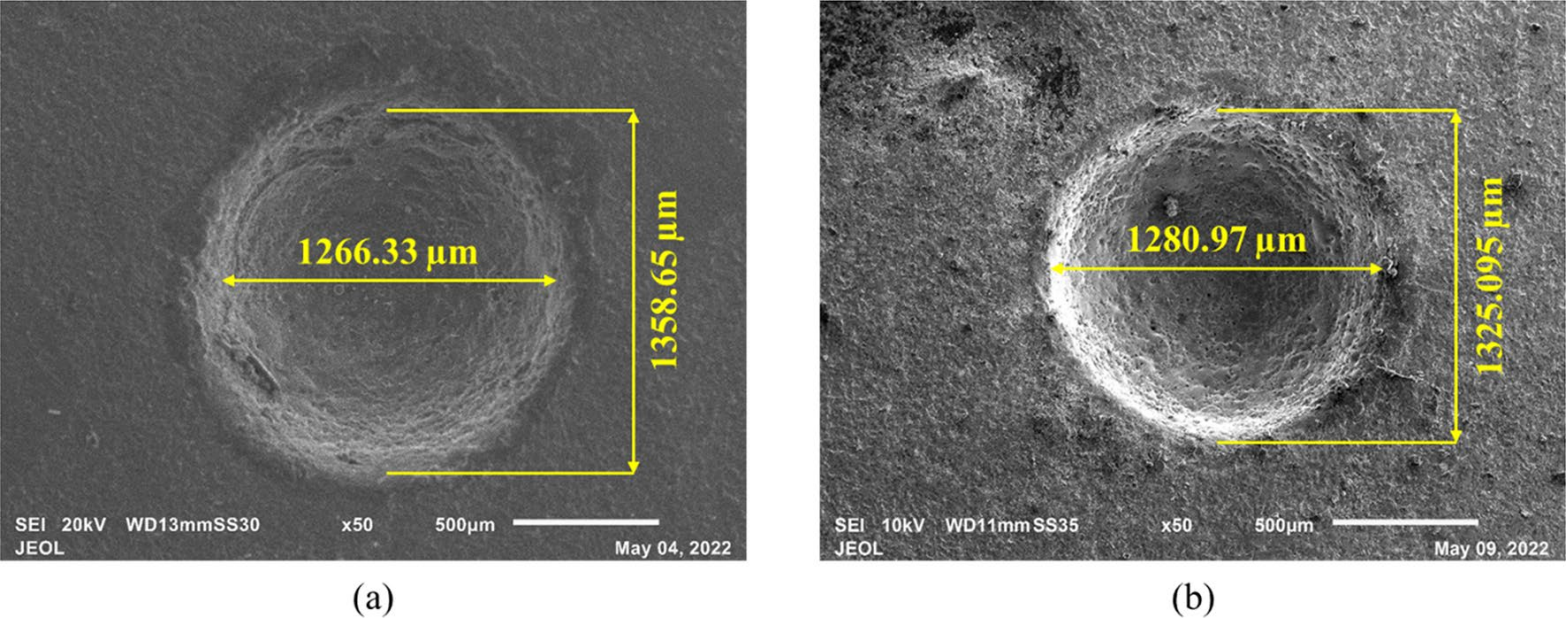
Micrographs of the machined microholes at (**a**) 70 V, 20 wt%, 50% DF and (**b**) 70 V, 30 wt%, 50% DF.

The sonication of electrolytes helps flush debris out and circulate fresh electrolytes at the machining zone. The increase in electrolyte concentration increased the number of conducting ions, thus, increasing the spark intensity. The aiding of sonication to the system increased penetration depth, which eventually resulted in through-hole formation. As shown in Fig. 8, the plots elucidate the effects of electrolyte concentration on the machining characteristics. The increase in concentration from 20 to 30 wt% enhanced all machining attributes. The surge in MRR, OC, and circularity can be attributed to the increase in anodic dissolution and intensity of the spark because of an increase in the number of conducting ions in the machining zones vicinity. The rise in OC and circularity can be justified by the SEM images shown in Fig. 9. It can be observed from Fig. 9a and b, that with a surge in concentration from 20 to 30 wt%, keeping voltage and duty factor constant at 70 V and 50% DF, the percent increase in OC and circularity is found to be 3.13%, and 3.71%, respectively. The average percentage surge in MRR, OC, and circularity with increased concentration is 42.45%, 12.52%, and 3.73%, respectively.

### Influence of duty factor on machining characteristics

**Figure 10:**
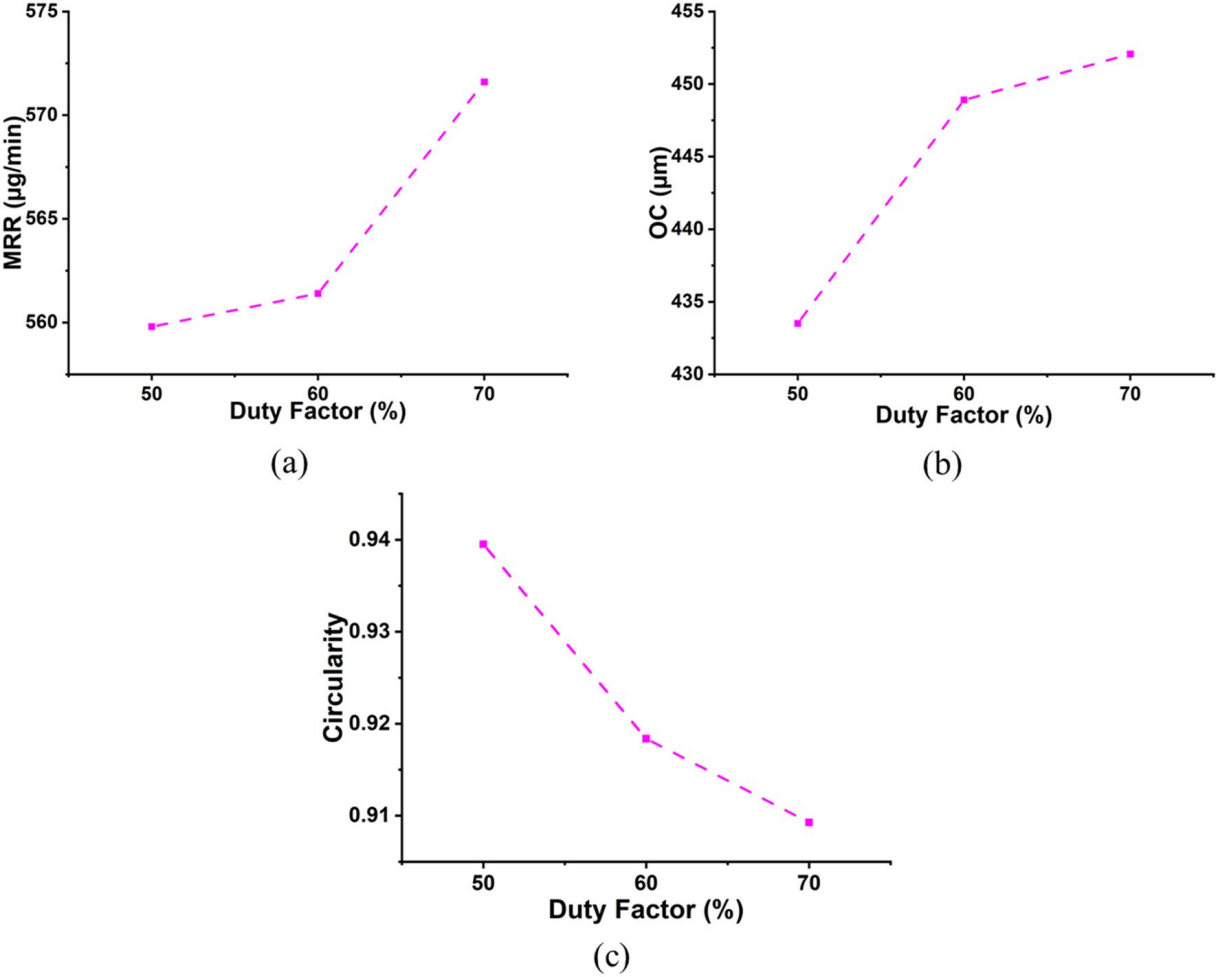
Influence of duty factor on machining characteristics (**a**) MRR, (**b**) OC, and (**c**) Circularity.

**Figure 11:**
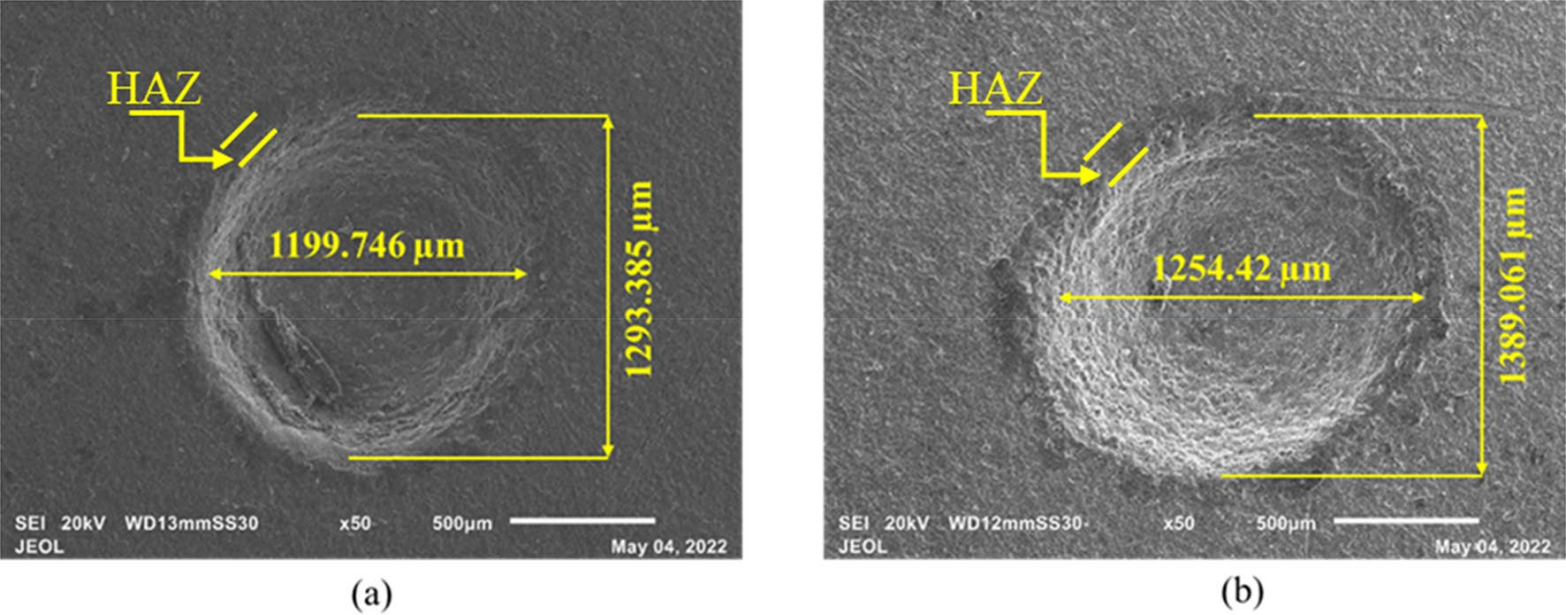
Micrographs of the machined microholes at (**a**) 70 V, 25 wt%, 50% DF and (**b**) 70 V, 25 wt%, 70% DF.

The duty factor is one of the critical parameters in machining, as proper tuning helps produce better-machined surfaces. Continuous DC lacks the ability of electrolyte replenishment in the machining zone, enabling increased thermal damage and heat-affected zones on the workpiece^53^. At low machining voltages, pulsing DC is responsible for making smooth surfaces. The plots in Fig. 10 indicate the influence of the duty factor on the machining attributes. The higher duty factor boosted MRR and OC while decreasing circularity. The rise in MRR and OC might be attributed to the existence of a discharge for a longer time, resulting in greater material removal at the surface leading to higher OC. The reduction in circularity of the machined microholes might be attributed to a longer pulse on time, which does not provide enough time for the molten material to cool, causing it to adhere to the machined hole’s surfaces and resulting in irregular micro holes. The average percentage surge in MRR and OC due to a rise in duty factor is 2.10% and 4.27%, respectively. The average percent reduction in circularity is found to be 3.59%. This percentage increase in OC and reduction in circularity can be justified by the SEM images shown in Fig. 11, and it can be observed that with an surge in duty factor from 50% DF to 70% DF keeping voltage and concentration constant at 70 V and 25 wt%, the percentage rise in OC is 13.67%, and the percentage reduction in circularity is found to be 4.14%. The increase in duty factor also increased the HAZ as the duration of the discharge is more.

**Figure 12:**
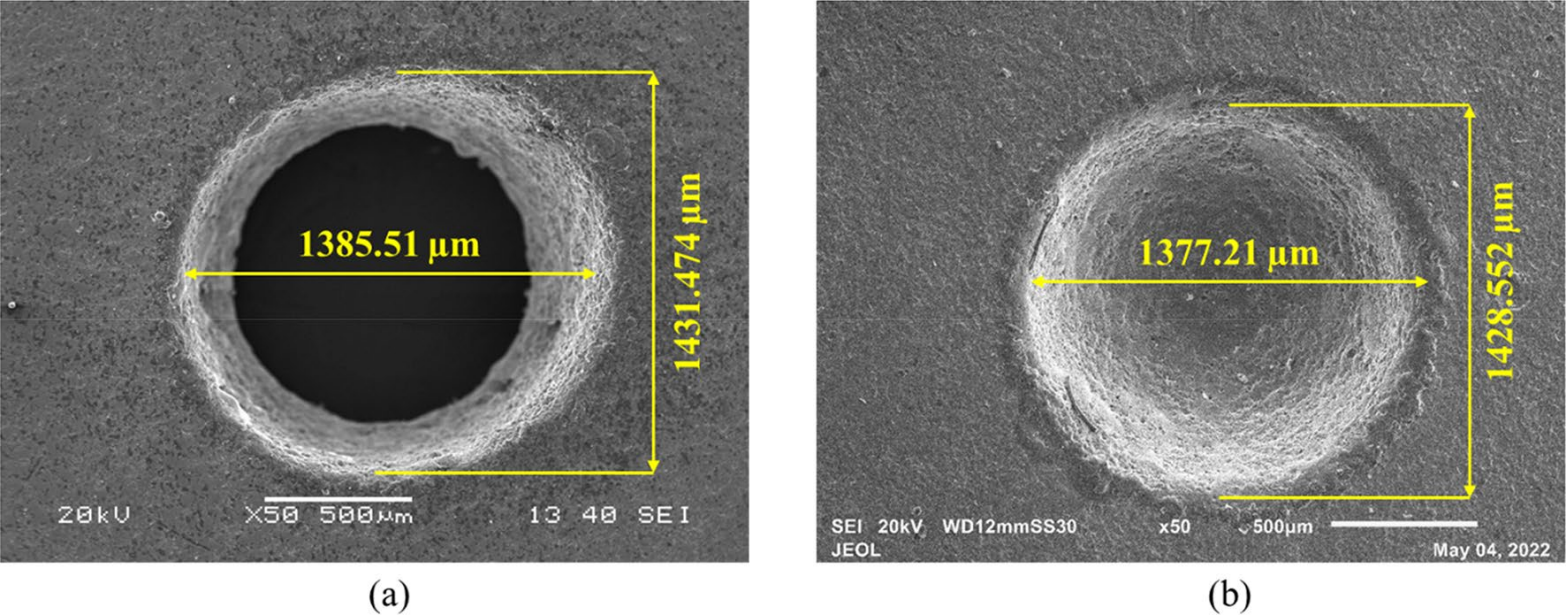
Micrographs of the machined microhole at (**a**) 80 V, 30 wt%, and 50% DF and (**b**) 80 V, 30 wt%, and 70% DF.

From the above discussion, it can be established that voltage is the major factor, followed by concentration for the machining characteristics like MRR and OC. Nevertheless, the circularity of the machined microholes is influenced by all the parameters with an edge-to-duty factor. The latter influences the circularity and is more effective than voltage and concentration. The SEM image shown in Fig. 12 elucidate that at 80 V, 30 wt%, and 50% DF through-hole was obtained, but at 80 V, 30 wt%, and 70% DF, a through-hole was not obtained, which might be due to a higher tool wear rate (axial wear). It is also found that the circularity of the machined hole decreased with an increase in duty factor. The circularity of the hole machined at 50% DF, keeping voltage and concentration at 80 V and 30 wt%, is higher than at 70% DF. The ultrasonication of the electrolyte helped in the proper replenishment of electrolyte from the machining zone and the successful evacuation of the debris formed. The channelization of the spark enabled uniform machining and the SEM micrographs shown in this article clearly show that no such protrusions are formed.

### Mathematical modeling and optimization

For the regression study, mathematical modeling was done using Design Experts-13 statistical software. The RSM modeling found quadratic relationships between the process parameters and the machining characteristics. Equations (4)–(6) are the respective regression equations for MRR, OC, and Circularity. The ANOVA results of the machining characteristics are given in Table 3, and it can be observed that voltage and concentration are significant for all the machining characteristics. However, the duty factor is significant for circularity and not MRR and OC. This statement agrees with the statement given at the end of the results and discussion. The ANOVA table (Table 3) suggests model adequacy for all the machining characteristics as the model is significant and lack of fit is not significant, which is desirable. The R^2^ and Adj. R^2^ value for MRR, OC, and circularity are 0.9815, 0.9648, 0.9790, and 0.9601, 0.9916, and 0.9840, respectively. These values are above 95%, which supports the model adequacy.

**Table 3:** ANOVA of the machining characteristics.

Response	Source	SS	DOF	MS	F-value	Prob > F	
MRR	Model	3.441E+05	9	38,232.13	58.83	< 0.0001	Significant
	Voltage	3.154E+05	1	3.154E+05	485.35	< 0.0001	Significant
	Concentration	21,068.10	1	21,068.10	32.42	0.0002	Significant
	Duty factor	348.10	1	348.10	0.5356	0.4810	Not significant
	Residual	6498.78	10	649.88			
	Lack of fit	4647.28	5	929.46	2.51	0.1677	Not significant
	Pure error	1851.50	5	370.30			
	Cor total	3.506E + 05	19				
OC	Model	1.260E+05	9	14,000.32	51.74	< 0.0001	Significant
	Voltage	1.154E+05	1	1.154E + 05	426.56	< 0.0001	Significant
	Concentration	5510.76	1	5510.76	20.37	0.0011	Significant
	Duty factor	860.14	1	860.14	3.18	0.1049	Not significant
	Residual	2705.68	10	270.57			
	Lack of fit	1932.11	5	386.42	2.50	0.1689	Not significant
	Pure error	773.57	5	154.71			
	cor total	1.287E+05	19				
Cir	Model	0.0147	9	0.0016	131.02	< 0.0001	Significant
	Voltage	0.0034	1	0.0034	275.74	< 0.0001	Significant
	Concentration	0.0029	1	0.0029	233.30	< 0.0001	Significant
	Duty factor	0.0029	1	0.0029	230.02	< 0.0001	Significant
	Residual	0.0001	10	0.0000			
	Lack of fit	0.0001	5	0.0000	0.6902	0.6530	Not significant
	Pure error	0.0001	5	0.0000			
	Cor total	0.0149	19				
MRR	R^2^ = 0.9815, adjusted R^2^ = 0.9648, predicted R^2^ = 0.8213, adequate precision = 27.0164						
OC	R^2^ = 0.9790, adjusted R^2^ = 0.9601, predicted R^2^ = 0.8600, adequate precision = 24.7892						
Cir	R^2^ = 0.9916, adjusted R^2^ = 0.9840, predicted R^2^ = 0.9697, adequate precision = 42.0792						

### Regression equation

4 $$ MRR = - {18}.{3511 } - {31}.{494}*A + 22.4743*B + 21.4366*C + 0.36375*A*B - 0.086875*A*C - 0.22625*B*C + 0.324091*A^{2} - 0.503636*B^{2} - 0.0759091*C^{2} $$

5 $$ OC = - 1371.29 + 32.5858*A - 45.5371*B + 26.1107*C + 0.038775*A*B - 0.0080625*A*C - 0.250075*B*C - 0.159488*A^{2} + 1.25045*B^{2} - 0.153058*C^{2} $$

6 $$ Cir = 1.10121 + 0.0343182*A - 0.0710065*B - 0.0184602*C + 7.97998e - 05*A*B + 0.000116855*A*C + 5.60122e - 05*B*C - 0.0002962*A^{2} + 0.0013095*B^{2} + 5.98704e - 05*C^{2} $$

where A, B, and C represent voltage, concentration, and duty factor, respectively.

### MOJAYA Optimization coupled with R-method (MADM)

**Figure 13:**
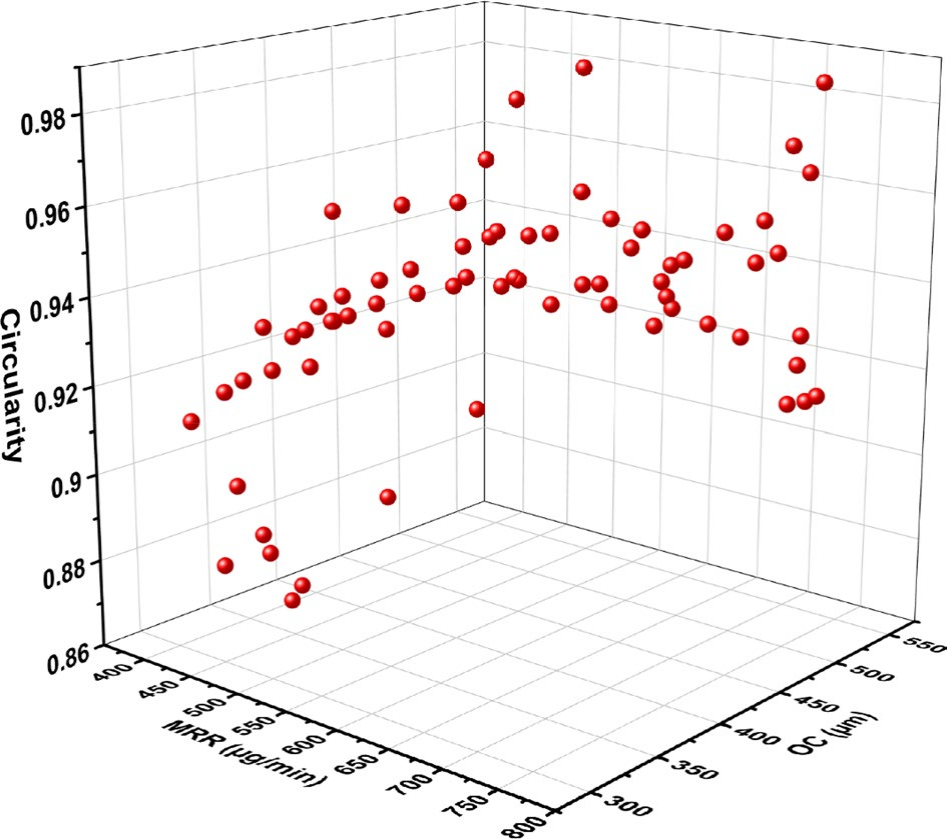
Pareto-optimal solutions obtained by the MO-JAYA algorithm.

The regression equation obtained from the RSM modeling is used as the objective function to perform optimization using the JAYA algorithm. The criterion selected for optimization was maximization of MRR and circularity and minimization of OC. A MATLAB code was developed for performing the MOJAYA optimization and validated with Rao et al.^54^. The Pareto optimum front was obtained using the posterior version of the optimization. As illustrated in Fig. 13, the Pareto optimal front for MRR, OC, and circularity objectives. A population size of 100 and an iteration or termination criteria of 100 were the parameter values utilized to generate 70 Pareto optimal solutions. MOJAYA algorithm does not have any algorithm-specific parameters that have to be tuned to reach a global optimum. This advantage of MOJAYA helps the algorithm find a global optimum instead of ending up in a local optimum^55^. The 70 Pareto optimal solutions obtained are all optimal as they are non-dominated solutions. To find an optimal value, different MADM techniques can be used, such as VIKOR^56^, TOPSIS^57^, AHP^58^, GRA^59^, PROMETHEE^60^ and R-method^61^. This article uses R-method to find optimal parameter among the generated Pareto optimal solution.

The responses are ranked based on the criterion of maximizing and minimizing. If the criteria are maximization, then the responses are ranked in decreasing order, and if the criteria are minimized, the responses are ranked in increasing order. The objective ranking is solely dependent on the decision-maker and which parameter is more useful for a particular application. The circularity is considered a major response, ranked 1, trailed by OC and MRR, ranked 2 and 3, respectively. A MATLAB code is developed for R-method and validated by Rao et al.^61^. Equation (7) is used to estimate the weights of the objectives and responses. Once weights are obtained for both objectives and responses, the composite score is calculated by summating the product of weights of objectives and responses. Finally, the row corresponding to the highest composite score is ranked one, followed by the others in decreasing order. Table 4 shows the Pareto optimal solutions, responses, weight calculations, composite score, and rank. The process parameters for rank “1” are considered to be optimal.

**Table 4:** Ranking of Pareto optimal solutions using R-method.

S.no.	Voltage (V)	Concentration (wt%)	Duty factor (%)	MRR (µg/min)	OC (µm)	Cir	W_MRR_	W_OC_	W_cir_	Composite score	Rank
1	80	24.9121	50.0000	761.3115	512.8938	0.9177	0.0126	0.0032	0.0050	0.0208	4
2	80	24.4613	50.0000	754.3719	509.8286	0.9164	0.0084	0.0033	0.0049	0.0166	10
3	79.09907	25.4128	50.0000	746.2855	509.8731	0.9241	0.0069	0.0033	0.0051	0.0152	15
4	80	23.7187	50.0000	742.4927	505.8869	0.9154	0.0061	0.0033	0.0049	0.0143	21
5	78.26752	26.3236	51.9508	740.1920	518.4086	0.9297	0.0055	0.0032	0.0051	0.0139	25
6	76.02681	30.0000	50.0000	723.5711	549.1111	0.9835	0.0051	0.0032	0.0154	0.0237	2
7	76.29447	29.0579	52.5397	722.6179	540.2913	0.9640	0.0049	0.0032	0.0094	0.0175	8
8	76.96309	27.4838	50.0000	720.9764	515.1542	0.9477	0.0046	0.0032	0.0063	0.0142	22
9	76.23098	27.9184	50.0000	708.3216	514.8684	0.9544	0.0045	0.0032	0.0077	0.0153	13
10	77.61558	25.2566	50.0000	708.2237	496.4462	0.9297	0.0043	0.0033	0.0051	0.0128	45
11	75.58334	29.3401	52.5981	708.2013	538.8408	0.9695	0.0042	0.0032	0.0111	0.0185	6
12	76.36989	27.2679	51.1664	705.4844	510.7709	0.9452	0.0041	0.0033	0.0062	0.0135	29
13	76.76797	25.1432	50.0000	686.8486	488.3049	0.9323	0.0040	0.0033	0.0053	0.0126	54
14	75.47756	27.4810	50.0000	685.9409	501.8420	0.9518	0.0039	0.0033	0.0071	0.0143	20
15	74.97059	26.6583	50.0000	665.1364	486.8137	0.9458	0.0038	0.0033	0.0063	0.0134	32
16	75.72253	25.1462	50.0000	663.1275	479.0898	0.9355	0.0037	0.0034	0.0054	0.0125	57
17	75.31695	25.5009	50.0000	658.7284	478.3241	0.9380	0.0037	0.0034	0.0056	0.0126	49
18	74.75761	26.4435	50.0000	657.8743	482.4064	0.9447	0.0036	0.0034	0.0061	0.0131	38
19	74.86871	25.9237	50.0000	654.1010	478.0679	0.9411	0.0036	0.0034	0.0059	0.0129	40
20	76.2625	23.5143	50.0000	651.8214	474.4994	0.9313	0.0035	0.0035	0.0052	0.0122	66
21	73.40903	27.1116	50.0000	635.7513	477.3564	0.9520	0.0035	0.0034	0.0073	0.0142	24
22	73.46057	26.6373	50.0000	631.7376	472.1625	0.9482	0.0034	0.0035	0.0065	0.0134	33
23	74.26935	24.4224	50.4634	622.5598	462.5972	0.9357	0.0034	0.0035	0.0054	0.0123	65
24	73.51852	25.2648	50.0000	616.8362	459.4008	0.9404	0.0033	0.0035	0.0057	0.0126	51
25	71.69478	27.7483	52.3659	608.5282	475.4719	0.9535	0.0033	0.0034	0.0074	0.0142	23
26	73.14622	25.0692	50.0000	606.6318	454.2501	0.9402	0.0033	0.0036	0.0057	0.0125	55
27	71.10073	27.8194	50.0000	594.2289	463.3313	0.9598	0.0032	0.0035	0.0089	0.0156	12
28	72.27736	24.4235	51.6937	584.0465	447.8304	0.9351	0.0032	0.0036	0.0053	0.0122	67
29	70.92579	26.8521	50.0000	581.5573	448.8294	0.9510	0.0032	0.0036	0.0068	0.0136	28
**30**	**69.36779**	**29.9294**	**50.0000**	**575.3300**	**480.0907**	**0.9858**	**0.0032**	**0.0034**	**0.0231**	**0.0296**	**1**
31	70.31554	26.8178	50.0000	569.1996	441.8967	0.9505	0.0031	0.0036	0.0067	0.0135	30
32	71.30196	24.9443	50.0000	568.0828	434.6844	0.9409	0.0031	0.0037	0.0058	0.0126	50
33	71.07342	25.1159	50.0000	565.6794	433.5527	0.9415	0.0031	0.0037	0.0060	0.0128	43
34	71.50956	23.7490	50.0000	557.0146	430.1196	0.9394	0.0031	0.0037	0.0056	0.0124	64
35	69.17648	27.0667	50.0000	549.7009	432.3870	0.9513	0.0030	0.0037	0.0070	0.0137	27
36	69.05519	26.9379	50.0000	546.2505	429.4639	0.9501	0.0030	0.0038	0.0066	0.0134	34
37	67.34873	29.8798	50.7548	536.8597	457.8526	0.9787	0.0030	0.0035	0.0126	0.0191	5
38	68.25802	26.6074	70.0000	536.1032	427.4037	0.9102	0.0030	0.0038	0.0049	0.0117	70
39	70.93583	22.7935	50.0000	533.2103	421.3819	0.9410	0.0030	0.0039	0.0059	0.0127	48
40	68.33048	26.8249	50.0000	531.8429	419.9254	0.9481	0.0029	0.0039	0.0064	0.0133	37
41	69.46429	24.7157	50.0000	530.8201	413.4796	0.9396	0.0029	0.0039	0.0057	0.0125	59
42	67.01361	29.1441	51.1823	527.0547	441.6361	0.9659	0.0029	0.0036	0.0101	0.0167	9
43	67.06317	28.2119	50.3081	520.6530	424.7208	0.9571	0.0029	0.0038	0.0085	0.0152	14
44	68.42478	24.5717	50.0000	510.6562	400.9902	0.9380	0.0029	0.0040	0.0055	0.0124	63
45	66.92044	26.5722	50.0000	504.6335	400.6338	0.9431	0.0029	0.0040	0.0061	0.0130	39
46	65.56996	25.4688	69.1501	490.1502	392.4421	0.8904	0.0029	0.0041	0.0049	0.0118	69
47	66.0712	25.9790	52.4441	490.0707	392.6835	0.9295	0.0028	0.0041	0.0051	0.0120	68
48	66.84156	25.0905	50.0000	489.2668	386.1352	0.9358	0.0028	0.0042	0.0055	0.0125	56
49	65.88606	26.6777	50.0000	488.0614	389.3599	0.9408	0.0028	0.0042	0.0058	0.0128	42
50	64.79864	28.8651	50.5735	486.1494	408.0703	0.9562	0.0028	0.0040	0.0082	0.0149	16
51	65.98694	25.0298	50.0000	474.7028	375.4685	0.9332	0.0028	0.0044	0.0053	0.0125	58
52	66.00457	24.2262	50.0000	466.6570	370.7712	0.9320	0.0028	0.0045	0.0052	0.0125	60
53	65.13689	25.5599	50.0000	466.2744	369.1692	0.9321	0.0028	0.0046	0.0053	0.0126	53
54	67.04869	22.5976	50.1279	464.3421	378.7209	0.9369	0.0028	0.0043	0.0055	0.0126	52
55	63.644	27.1498	50.0986	456.1479	367.0865	0.9351	0.0027	0.0046	0.0054	0.0128	44
56	64.83383	24.0391	51.9291	451.7704	363.1898	0.9215	0.0027	0.0047	0.0050	0.0125	61
57	65.36465	23.7089	50.0000	451.1217	360.6896	0.9301	0.0027	0.0048	0.0052	0.0127	46
58	62.83773	24.2176	68.3147	447.7099	357.2630	0.8706	0.0027	0.0050	0.0048	0.0125	62
59	62.13306	29.6190	50.0927	446.5021	385.0164	0.9549	0.0027	0.0043	0.0079	0.0149	18
60	63.3882	26.3816	50.0000	446.4681	354.7008	0.9289	0.0027	0.0051	0.0051	0.0129	41
61	62.37995	24.9836	69.1949	445.9452	350.8380	0.8676	0.0027	0.0053	0.0048	0.0127	47
62	63.32197	25.2303	50.5469	437.4625	345.6659	0.9215	0.0027	0.0057	0.0050	0.0134	35
63	60.97693	26.3690	61.6789	432.2748	344.8875	0.8787	0.0027	0.0059	0.0048	0.0134	31
64	61.30145	28.0105	50.0000	426.7813	347.0244	0.9307	0.0027	0.0054	0.0052	0.0133	36
65	60	27.8428	61.9266	425.2203	344.8178	0.8826	0.0026	0.0063	0.0048	0.0138	26
66	62.58623	25.1440	50.0000	424.8975	333.2722	0.9197	0.0026	0.0067	0.0050	0.0144	19
67	61.68872	23.9978	55.1974	416.3976	332.9004	0.8949	0.0026	0.0074	0.0049	0.0149	17
68	61.27419	26.3128	50.0000	416.0564	325.8303	0.9172	0.0026	0.0084	0.0050	0.0160	11
69	60.34578	24.9195	58.3517	413.1327	324.8162	0.8768	0.0026	0.0103	0.0048	0.0177	7
70	60	26.6436	50.0000	401.5340	311.6440	0.9111	0.0026	0.0154	0.0049	0.0229	3

Significant values are in bold.

**Figure 14:**
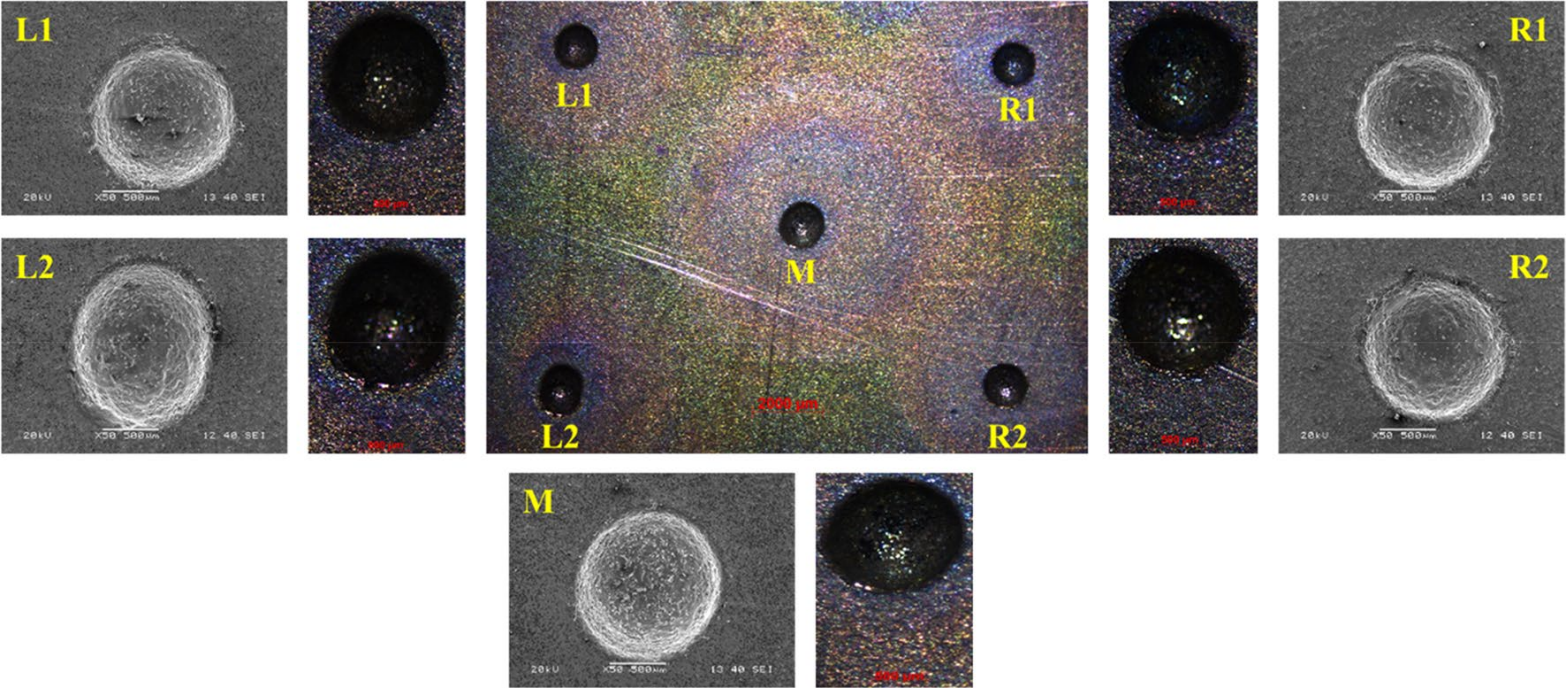
Optical microscope and SEM micrographs of the machined multiple microholes at the optimal process parameter setting of 69 V, 30 wt%, and 50% DF.

It can be observed that the optimal process parameters obtained by the integrated MOJAYA algorithm and R-method are 69.36779 V, 29.9294 wt%, and 50% DF. The predicted values are rounded to the nearest integer, and validation tests are conducted to check the feasibility of the results obtained. The process parameters are rounded to 69 V, 30 wt%, and 50% DF, and experiments were conducted five times. The average values of the machining characteristics are tabulated in Table 5. It can be seen that the predicted and the experimental values are in close agreement with each other, with an error percent not exceeding ± 5. The SEM image obtained for the experiment conducted at the optimal parameter setting is shown in Fig. 14. The SEM micrograph suggests that the machined microholes obtained at optimal process parameters are smooth with high circularity and minimal OC.

7 $$ W_{i} = \frac{{\left( {\frac{1}{{\sum\limits_{a = 1}^{i} {\left( {\frac{1}{{x_{a} }}} \right)} }}} \right)}}{{\sum\limits_{i = 1}^{j} {\left( {\frac{1}{{\sum\limits_{a = 1}^{i} {\left( {\frac{1}{{x_{a} }}} \right)} }}} \right)} }} $$

where, ${x}_{a}$: rank of the alternatives/objectives a ($a=\mathrm{1,2},3,\dots ,i$), $j$: number of alternatives or objectives, ${W}_{i}$: weight of the alternatives/objectives $i$ ($i=\mathrm{1,2},3,\dots ,j$).

**Table 5:** Validation test results.

	Responses		
	MRR (mg/min)	OC (µm)	Circularity
Actual (69 V, 30 wt%, 50% DF)	590.5	494.683	0.9615
Predicted (69.36779 V, 29.9294 wt%, 50% DF)	575.3300	480.0907	0.9858
Error (±)	2.63%	3.039%	2.46%

## Conclusions

In this article, the experimentation was conducted on a commercially pure titanium plate of thickness 1000 µm using a titanium tool of diameter 800 µm for machining microholes using a tailor-made ES-µ-ECDM system characterizing the machining responses like MRR, OC, and circularity. Further, multi-objective optimization is accomplished using the MOJAYA algorithm, and the optimal process parameter value is predicted using R-method (MADM). The following conclusion is drawn from this study:

- The ultrasonication of the electrolyte helped stabilize the spark by improving the machining depth with an increase in process parameters. Through holes were obtained at two parameter settings of 80 V, 25 wt%, and 60% DF and 80 V, 30 wt%, and 50% DF.
- The ultrasonication of the electrolyte enabled uniform machining by channelizing the spark at the machining zone even at higher depths (> 300 µm) and produced protrusion-free surfaces.
- The assistance of ultrasonication helped in proper flushing of the debris from the machining zone and replenishes the electrolyte which established a stable gas film around the tool surface which enhanced the depth of penetration.
- The rise in voltage and concentration increased all machining characteristics, but with a rise in duty factor, the MRR and OC increased, and the circularity reduced.
- The ANOVA of the machining characteristics suggested that the duty factor helps in improving surface characteristics like circularity.
- The 70 Pareto-optimal solutions were acquired by performing the posterior version of metaheuristic optimization. The optimal process parameter was obtained using R-method, where circularity was considered the prime objective, followed by OC and MRR.
- The optimal value predicted was 69.36779 V, 29.9294 wt%, 50% DF, which was rounded to 69 V, 30 wt%, and 50% DF to perform the experiment, and the results obtained at these parameter settings are in good agreement with the error percent not exceeding ± 5%.

The authors suggest that the optimal process parameter obtained can provide a better microhole with minimal overcut and maximum circularity. Hence micromachining of commercially pure Titanium can be achieved using ES-µ-ECDM.

## Data Availability

The datasets used and/or analyzed during the current study are available from the corresponding author on reasonable request.
